# Associations among MHC genes, latitude, and avian malaria infections in the rufous‐collared sparrow (*Zonotrichia capensis*)

**DOI:** 10.1002/ece3.11634

**Published:** 2024-07-17

**Authors:** Juan Rivero de Aguilar, Omar Barroso, Elisa Bonaccorso, Hector Cadena, Lucas Hussing, Josefina Jorquera, Javier Martinez, Josué Martínez‐de la Puente, Alfonso Marzal, Fabiola León Miranda, Santiago Merino, Nubia E. Matta, Marilyn Ramenofsky, Ricardo Rozzi, Carlos E. Valeris‐Chacín, Rodrigo A. Vásquez, Juliana A. Vianna, John C. Wingfield

**Affiliations:** ^1^ Centro Subantártico Cabo de Hornos (CHIC) Universidad de Magallanes Puerto Williams Chile; ^2^ Departamento de Ciencias Ecológicas, Facultad de Ciencias Universidad de Chile Santiago Chile; ^3^ Instituto de Ecología y Biodiversidad, Facultad de Ciencias Universidad de Chile Santiago Chile; ^4^ Laboratorio de Biología Evolutiva, and Instituto Biósfera, Colegio de Ciencias Biológicas y Ambientales Universidad San Francisco de Quito Quito Ecuador; ^5^ Centro de la Biodiversidad y Cambio Climático Universidad Tecnológica Indoamérica Quito Ecuador; ^6^ Departamento de Ecología, Instituto Para el Desarrollo Sustentable, Facultad de Ciencias Biológicas Pontificia Universidad Católica de Chile Santiago Chile; ^7^ Departamento de Biomedicina y Biotecnología (Area de Parasitología) Universidad de Alcalá de Henares Madrid Spain; ^8^ Departamento de Parasitología Universidad de Granada Granada Spain; ^9^ Ciber de Epidemiología y Salud Pública (CIBERESP) Madrid Spain; ^10^ Departamento de Anatomía, Biología Celular y Zoología, Facultad de Biología Universidad de Extremadura Badajoz Spain; ^11^ Grupo de Investigaciones en Fauna Silvestre Universidad Nacional de San Martín Tarapoto Peru; ^12^ Departamento de Ecología Evolutiva Museo Nacional de Ciencias Naturales CSIC Madrid Spain; ^13^ Departamento de Biología, Facultad de Ciencias Universidad Nacional de Colombia Bogotá Colombia; ^14^ Department of Neurobiology, Physiology and Behavior University of California Davis California USA; ^15^ Millennium Institute Center for Genome Regulation (CRG) Millennium Institute of Biodiversity of Antarctic and Subantarctic Ecosystems (BASE), Millennium Nucleus of Patagonian Limit of Life (LiLi) Santiago Chile

**Keywords:** haemosporidian parasites, major histocompatibility complex, parasite‐mediated selection

## Abstract

The major histocompatibility complex (MHC) is a genetic region in jawed vertebrates that contains key genes involved in the immune response. Associations between the MHC and avian malaria infections in wild birds have been observed and mainly explored in the Northern Hemisphere, while a general lack of information remains in the Southern Hemisphere. Here, we investigated the associations between the MHC genes and infections with *Plasmodium* and *Haemoproteus* blood parasites along a latitudinal gradient in South America. We sampled 93 rufous‐collared sparrows (*Zonotrichia capensis*) individuals from four countries, Colombia, Ecuador, Peru, and Chile, and estimated MHC‐I and MHC‐II allele diversity. We detected between 1–4 (MHC‐I) and 1–6 (MHC‐II) amino acidic alleles per individual, with signs of positive selection. We obtained generalized additive mixed models to explore the associations between MHC‐I and MHC‐II diversity and latitude. We also explored the relationship between infection status and latitude/biome. We found a non‐linear association between the MHC‐II amino acidic allele diversity and latitude. Individuals from north Chile presented a lower MHC genetic diversity than those from other locations. We also found an association between deserts and xeric shrublands and a lower prevalence of *Haemoproteus* parasites. Our results support a lower MHC genetic in arid or semi‐arid habitats in the region with the lower prevalence of *Haemoproteus* parasites.

## Graphical abstract

We investigated MHC gene associations with *Plasmodium* and *Haemoproteus* infections in South American rufous‐collared sparrows across a latitudinal gradient, finding non‐linear associations between MHC‐II diversity and latitude, with individuals from north Chile exhibiting lower genetic diversity. Additionally, we observed lower *Haemoproteus* prevalence in desert and xeric shrubland habitats, supporting a link between MHC genetic diversity and parasite prevalence in arid environments.

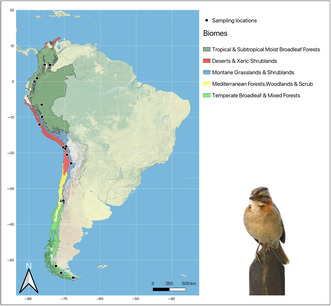

## 1 INTRODUCTION

The major histocompatibility complex (MHC) is a genetic region in jawed vertebrates harboring key immune genes involved in the immune response (Kaufman, 2018). Among them, the classical MHC‐I genes and MHC‐II genes code for proteins that conform to the so‐called “MHC molecules” (Martin & Kaufman, 2022), which are involved in the presentation of parasite antigens (in the form of short peptides) to T lymphocytes (Rock et al., 2016). If peptides are recognized as non‐self, it triggers the activation of cytotoxic T cells or switching on B cells to produce antibodies (Radwan et al., 2020). Because of this key role in immune defenses, MHC genes have been described as the “center of the immunological universe” (Trowsdale, 1995). MHC‐I molecules mainly present peptides from intracellular antigens, in contrast, MHC‐II present those from extracellular ones (Hess & Edwards, 2002).

Depending on the species, avian MHC genes have been found being both polygenic (one to several loci) and polymorphic (multiple alleles at each locus). Individual MHC is characterized by the number of gene copies (specific to each species) (Minias et al., 2019; Westerdahl et al., 2022) and by the heterozygosity at each locus (Alcaide et al., 2008). These will determine the number of MHC molecules (or MHC diversity) expressed by an individual, and collectively that of an entire population. MHC genes are codominantly expressed (both alleles at each locus) (Murphy & Weaver, 2017); thus, depending on the MHC genotype, each individual will express different MHC molecules, varying in their ability to present antigens (Nikolich‐Zugich, 2004).

MHC are the most polymorphic genes found in vertebrates (Borghans et al., 2004), and this polymorphism results from the elevated variability observed in the peptide binding region (PBR), the cleft where peptides accommodate in the MHC molecule (Lenz, 2011). Different non‐exclusive mechanism have been proposed to explain MHC diversity (Edwards & Hedrick, 1998; Piertney & Oliver, 2006; Radwan et al., 2020; Spurgin & Richardson, 2010; van Oosterhout, 2009). Among them, the parasite‐mediated selection hypothesis states that MHC diversity is maintained by selective pressures caused by parasites (Piertney & Oliver, 2006). MHC should reflect the past and current selective pressures that a species (or population) has suffered over evolutionary time (Hasselquist, 2007; Levy et al., 2020; Minias et al., 2019; O'Connor et al., 2019). Within birds, passerines generally have more MHC gene copies than non‐passerines, evidencing different evolutionary histories (Minias et al., 2019; O'Connor et al., 2019). Having an elevated MHC allele diversity (heterozygous advantage hypothesis) or rare MHC alleles (rare allele advantage hypothesis) would increase the recognition and presentation of peptides from parasites to the immune system cells (Spurgin & Richardson, 2010). In turn, spatially heterogeneous selective pressures from parasites would maintain different MHC alleles at a local scale (Loiseau et al., 2009).

Associations between MHC and the occurrence of diseases have been found in humans and other vertebrates (Lundie et al., 2008; Sanchez‐Mazas, 2020), supporting the role of parasites in the evolution of MHC. Several resistance/susceptibility associations in birds have been observed in both experimental and field studies (reviewed in O'Connor et al., 2019), thus stimulating further research on the role of MHC in wild birds (Hasselquist, 2007; Minias et al., 2019; O'Connor et al., 2019). Due to its global distribution and relative ease of sampling, avian malaria parasites *Plasmodium* and *Haemoproteus* have become an excellent model for studying the ecology of host–parasite interactions in birds (Santiago‐Alarcon & Marzal, 2020). Parasites of the *Plasmodium* and *Haemoproteus* genera have a cosmopolitan distribution and include diverse molecular and morphological species (Clark et al., 2014). These parasites are blood protozoan parasites that commonly infect birds in the wild (Valkiūnas, 2005) and are transmitted by dipteran vectors of the Family Culicidae (*Plasmodium*), Ceratopogonidae (Subgenus *Parahaemoproteus*), and Hippoboscidae (Subgenus *Haemoproteus*) (Santiago‐Alarcon et al., 2012). Local extinctions of bird species have evidenced their impact on avian hosts (Atkinson et al., 2000), detrimental effects on individual fitness (Asghar et al., 2015; Merino et al., 2000), survival (Martínez‐de la Puente et al., 2010), and virulence (Videvall et al., 2020). However, infections are commonly detected as low parasitemia chronic infections with mild or not apparently detrimental effects on individuals (Asghar et al., 2011).

To test the parasite‐mediated selection hypothesis and its role in MHC allele diversity, we investigated the associations between MHC‐I and MHC‐II allele diversity and the prevalence of *Plasmodium* and *Haemoproteus* parasites in the rufous‐collared sparrow (*Zonotrichia capensis*) (P. L. Statius Müller, 1776) across a latitudinal gradient along South America. The rufous‐collared sparrow is a small passerine (16.8–31 g) found in open spaces from sea level to high elevation (~4600 m) (Rising & Jaramillo, 2023). Its distribution range spans Central and South America, from southern Mexico to Cape Horn in Chile. Numerous morphological subspecies have been proposed resulting from geographical differences in plumage, morphology and song (Chapman, 1940; Handford, 1985). All subspecies are mainly sedentary except for the southernmost species, the long‐distance migratory *Z. capensis australis*. However, short‐distance movements have been observed in resident subspecies related to altitudinal movements during winter/non‐winter seasons (Poblete et al., 2023; Rising & Jaramillo, 2023). The rufous‐collared sparrow is commonly infected by these parasites throughout its distribution range (Cadena‐Ortiz et al., 2019; Doussang et al., 2019; Jones et al., 2015; Mantilla et al., 2016).

Based on the decreasing latitudinal gradient in biological diversity (LGD) from the Equator to high latitudes (Willig et al., 2003), we hypothesize that birds from locations close to the Equator would present higher MHC allele diversity and higher parasite prevalence compared with birds from the austral region of South America. By suffering a higher selective pressure from parasites, individuals would have evolved a vast array of immune defenses, that is, MHC allele diversity (Biedrzycka et al., 2018; Demas & Nelson, 2012; Hasselquist, 2007; Møller, 1998; Owen‐Ashley et al., 2008). Previous studies have found evidence of a latitudinal gradient of *Plasmodium* and *Haemoproteus* infections in South America, with a general decreasing of both parasites to the south (Clark et al., 2014; Durrant et al., 2006; Fecchio et al., 2019; Merino et al., 2008; White et al., 1978). However, other studies have not observed this trend (Clark, 2018; Doussang et al., 2019). Thus, if avian malaria parasites do not follow a latitudinal gradient in prevalence, the MHC allele diversity would be lower in regions where parasite prevalence is low or absent (Hawley & Fleischer, 2012; Johnson & Haas, 2021). In this situation, biomes (Dinerstein et al., 2017; Olson et al., 2001) could represent a better approach to explain MHC diversity, since the biotic and abiotic factors of these biomes may affect the constitution of distinct assemblages of parasites, vectors, and hosts (Chapa‐Vargas et al., 2020; Cuevas et al., 2020; Doussang et al., 2019; Garcia‐Longoria et al., 2022; Hussing, 2020). In order to test this hypothesis, we specifically (i) estimated MHC‐I and MHC‐II allele diversity in rufous‐collared sparrows in a latitudinal gradient in South America (from Colombia to Cape Horn in Chile), (ii) explored signals of positive selection on MHC alleles, (iii) investigated the associations among MHC allele diversity and latitude/infection, and (iv) analyzed the associations among parasite infection status and latitude/biomes.

## 2 MATERIALS AND METHODS

### 2.1 Study area and bird sampling

**FIGURE 1:**
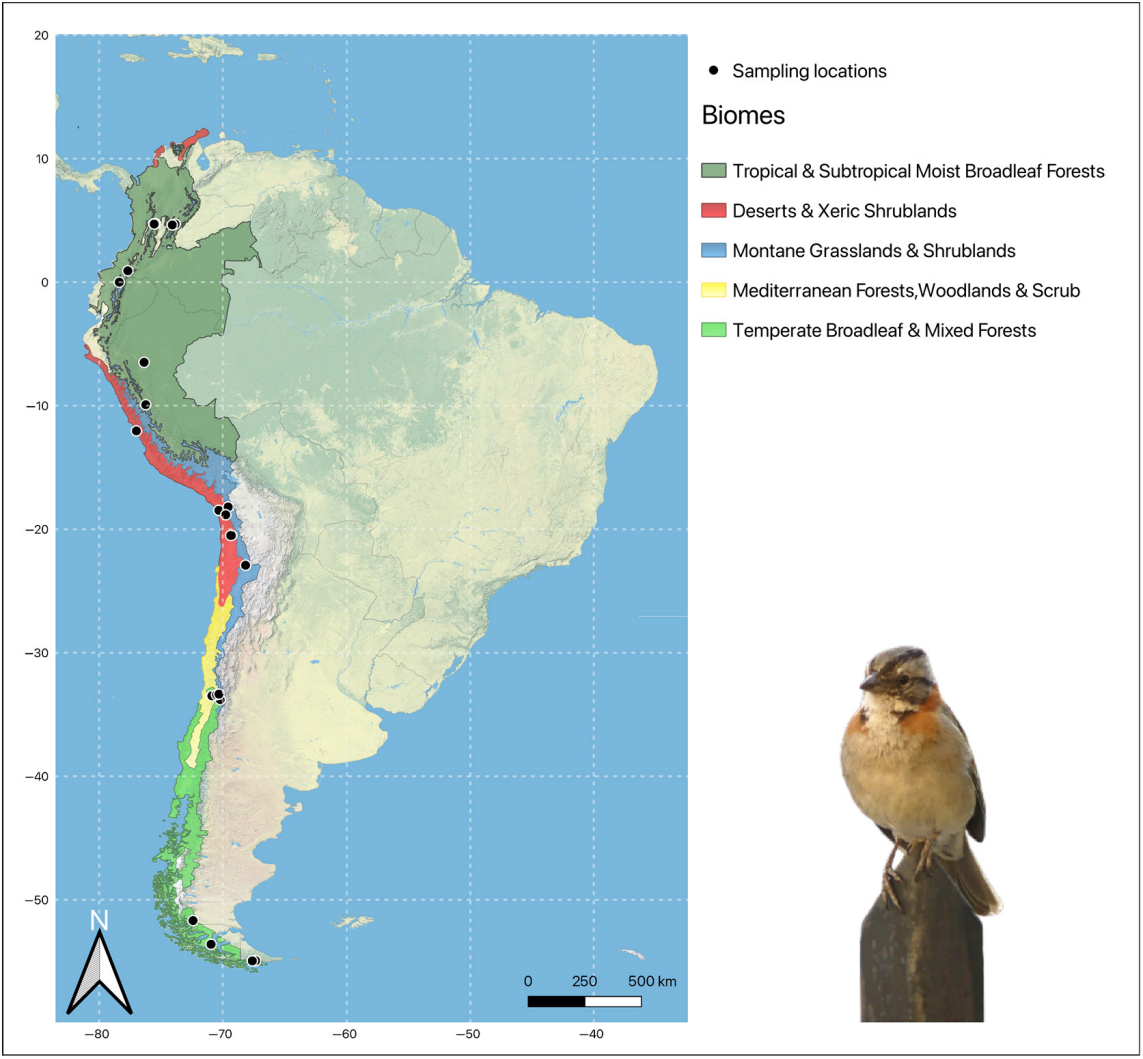
Sampling locations and biogeographical biomes of rufous‐collared sparrows. Biomes and map where obtained with QGIS.

We investigated MHC‐I and MHC‐II genes in 93 rufous‐collared from Colombia, Ecuador, Peru and Chile, sampled in different studies from 2011 to 2018 (Basto et al., 2006; Cadena, 2015; Cadena‐Ortiz et al., 2019; González et al., 2015; Hussing, 2020; Martínez et al., 2016; Marzal et al., 2015) (Figure 1, Table S1). Twenty‐one individuals were sampled in Colombia, 22 in Ecuador, 11 in Peru, and 39 in Chile.

### 2.2 MHC primes design, amplification, and sequencing

Genomic DNA was extracted by different methods depending on the laboratory of origin (Cadena, 2015; Cadena‐Ortiz et al., 2019; Doussang et al., 2019; González et al., 2015; Martínez et al., 2016; Marzal et al., 2015). In Chile, and before molecular analyses, DNA samples were quantified in a Qubit fluorometer (Thermo Fisher). A Sequencing Library Preparation protocol (Illumina) was applied for the Illumina MiSeq System to obtain MHC‐I and MHC‐II sequences. The workflow consisted of an initial MHC primer design (Macrogen) with an overhang adapter attached to MHC‐I and MHC‐II forward and reverse primers. For MHC‐I, we tested two different sets of primers, GCA21M (5′‐CGTACAGCGGCTTGTTGGCTGTGA‐3′) and fA23M (5′‐GCGCTCCAGCTCCTTCTGCCCATA‐3′) (Jones et al., 2014), and MhcPasCI‐FW (5′‐CSCSCAGGTCTSCACAC‐3′) and MhcPasCI‐RV (5′‐CWCARKAATTCTGYTCHCACC‐3′) (Alcaide et al., 2013). For MHC‐II, we tested two sets of primers, HOPE1 (5’‐GAAAGCTCGAGTGTCACTTCACGAACGGC‐3′) and HOPE 10 (5’‐TCCACGCTGAACGGGCGGAACACCTC‐3′) (Sato et al., 2011), and 2zffw1 (5’‐TGTCACTTCAYKAACGGCACGGAG‐3′) and 2zfrv1(5’‐GTAGTTGTGCCGGCAGTACGTGTC‐3′) (Balakrishnan et al., 2010). We tested primers with the overhang adapters in five individuals through a standard PCR (Sallaberry‐Pincheira et al., 2016). All primers worked successfully for the rufous‐collared sparrow, however, we finally selected GCA21M/fA23M and HOPE1/HOPE10 because they were applied before in this species successfully (Jones et al., 2014; Sato et al., 2011). The GCA21M/fA23M primers amplified a region of 214 base pairs (bp) inside exon 3 of the alpha chain, a part of the PBR, whereas HOPE1/HOPE10 amplified 189 bp inside exon 2 of the beta chain, also part of PBR. PCR conditions for GCA21M/fA23M consisted of 94°C for 10 min, 35 cycles of 94°C for 30 s, 64°C for 30 s, and 72°C for 1 min, and at 72°C for 10 min. PCR conditions for HOPE1/HOPE10 consisted of a PCR of 94°C for 10 min, 35 cycles of 94°C for 30 s, 58°C for 30 s, 72°C for 1 min, and 72°C for 10 min (Jones et al., 2014; Sato et al., 2011). Amplification success was confirmed by observing UV bands of appropriate size in an agar 1% gel stained with SYBR Green (Sigma Aldrich). The MHC‐I and MHC‐II amplicons were sequenced in Macrogen. We included a PCR mix reaction without a DNA template in every PCR run as the negative control.

### 2.3 Library preparation and sequencing

Purified PCR products in a final 30‐μL volume with MHC‐I and MHC‐II amplicons were quantified in Nanodrop. After the purification Nextera XT v2 Index Kit A indices were added (Illumina). After a second purification with AMPure (Beckman Coulter), the libraries were diluted in 10 nM before making the equimolar pool, to which a 10% PhiX control library (Illumina) was added. The entire pool was then diluted to 10 pM, which was loaded onto the MiSeq sequencer (Illumina) with the Kit v3 (600 Cycles) and mode of sequencing 2 × 300 pb (PE).

### 2.4 Bioinformatics

Each MHC‐I and MHC‐II FASTQ sequences were merged with the AmpliMERGE tool in AmpliSAT, and then, the quality was checked with AmpliCHECK (Sebastian et al., 2016). Clustering and filtering sequences were performed in AmpliSAS by selecting the Illumina platform. Elimination of chimeras and sequences with a length different from the expected sequence size was also chosen in this step (Rekdal et al., 2018). A total of 21.318.682 reads (59.5% quality score > =Q30) were obtained from sequencing. From them, we obtained the total number of MHC‐I and MHC‐II nucleotide sequences for every individual. MHC‐I and MHC‐II sequences were aligned in Bioedit (Hall, 2011) with CLUSTAL and translated into amino acids. Sequences with stop codons were considered non‐functional and removed from the dataset. Amino acidic MHC sequences were reversed to nucleotides and searched in Blast (https://blast.ncbi.nlm.nih.gov/Blast.cgi) to confirm whether they corresponded to new (unpublished) or known MHC nucleotide sequences. Blast matches lower than 100% were considered as new MHC alleles. We refer to the “MHC allele,” although primers do not discern among loci and do not cover all MHC‐I exon 3 and MHC‐II exon 2. MHC‐I and MHC‐II were considered all putative sequences even they came from one PCR event.

### 2.5 Phylogenetic reconstruction and selection in the peptide binding region

We combined nucleotide sequences from all individuals in two datasets (MHC‐I and MHC‐II), aligned with MUSCLE (Edgar, 2004), and applied redundancy sequence removal in Jalview (Waterhouse et al., 2009). A maximum likelihood phylogenetic tree for each MHC class plus other published rufous‐collared sparrow MHC‐I and MHC‐II sequences were obtained in NGPhylogeny.fr (Lemoine et al., 2019). We selected BMGE curation and PhyML tree inference. The best model of molecular evolution was estimated by SMS (Lefort et al., 2017) and node support with aBayes. Trees were edited in iTol (Letunic & Bork, 2021), and chicken *Gallus gallus* MHC‐I (GenBank accession number KM014730.1) and MHC‐II (GenBank accession number AY744349.1) were selected as outgroups.

To explore signs of positive selection operating on the MHC, we performed a selection analysis of MHC‐I and MHC‐II nucleotide sequences in the Datamonkey 2.0 server (Weaver et al., 2018) by mean of a Bayesian approach to infer non‐synonymous (dN) and synonymous (dS) substitution rates per‐site with FUBAR method (Murrell et al., 2013). Excess of non‐synonymous vs synonymous substitutions was considered a sign of positive selection. In addition, we calculated Tajima's D in the PBR versus the non‐PBR positions under selection following Minias et al. (2018) in MEGA11 (Tamura et al., 2021).

### 2.6 Individual parasite infection status

*Plasmodium* and *Haemoproteus* infection status were determined by amplifying the cytochrome b DNA by PCR, microscopy, or both techniques (see Table S1). Different combinations of primers were used in each laboratory of origin (Cadena, 2015; Cadena‐Ortiz et al., 2019; Doussang et al., 2019; González et al., 2015; Martínez et al., 2016; Marzal et al., 2015). Seventy‐three out of 93 individuals were also investigated by inspecting blood smears by microscopy (Cadena‐Ortiz et al., 2019; González et al., 2015; Hussing, 2020). The combined use of both methodologies enhanced the accuracy of infection confirmation, as different primers can produce slight variations in parasite detection (Valkiūnas et al., 2006, 2008).

### 2.7 Statistical analyses

#### 2.7.1 Associations between MHC allele diversity and latitude

Associations among MHC diversity and latitude were investigated by generalized additive mixed models (GAMM) with the mgcv package (Wood, 2023) in R (R Development Core Team, 2016). For each MHC class, we created a set of competitive models including MHC‐I or MHC‐II amino acidic allele diversity as dependent variable, and latitude, location, and year as explanatory variables. In the models, latitude was included as a smooth parameter = restricted maximum likelihood (REML). Because birds' sampling spanned different years and several individuals came from the same location, year and location were included as random effects. An AICc model selection (suited for small sample sizes) was performed over all model term combinations with family = Poisson (MHC‐I) or Gaussian (MHC‐II), based on previous data exploration of the dependent variable distribution. Model assumptions were checked with gam.check. We evaluated the goodness of fit of the final models by inspecting the dependence of the residuals, and checked their normality with qqplots. The final best model and also the equally possible models were obtained within a threshold of delta <2.

#### 2.7.2 Associations among infections and latitude/biomes

In order to explore whether infections follow the same pattern as the observed for MHC amino acidic allele diversity and latitude, we investigated the associations between infection status and latitude using logistic GAMM models. Both *Plasmodium* and *Haemoproteus* infection status (non‐infected = 0 and infected = 1) were selected as the dependent variables and latitude, location, and year as explanatory variables. As described previously, latitude was included as a fixed factor and year and location as random effects. An AICc model selection was performed over all models obtained with method = “REML” and family = binomial. Model assumptions were checked as before.

Finally, differences in *Plasmodium* and *Haemoproteus* prevalence of infection among biomes were investigated by Fisher exact tests with post hoc Bonferroni correction applied among factor levels. Biomes were obtained for every location by importing GPS data points into QGIS (QGIS.org, 2022) and loading a shapefile layer of “Biomes of the World” for each country (Dinerstein et al., 2017; Olson et al., 2001).

## 3 RESULTS

### 3.1 MHC allele diversity

The average number of reads ± [SD] per sample was 1273 ± 361 (MHC‐I) and 901 ± 198 (MHC‐II). We obtained 48 MHC‐I and 104 MHC‐II nucleotide sequence variants when considering all individuals (Tables S1 and S2). Individual MHC allele nucleotide diversity ranged from one to four alleles for MHC‐I (mean = 1.8, SD = 0.99), and from one to six for MHC‐II (mean = 3.34, SD = 1.21). When converted to amino acids, the MHC allele amino acidic diversity was similar (MHC‐I: mean = 1.57, SD = 0.87; MHC‐II mean = 3.28, SD = 1.16). A total of 21 MHC‐I stop codon sequences were removed from the dataset, while no stop codon sequences were observed for MHC‐II. Considering that MHC genes are codominantly expressed, the maximum number of MHC‐I alleles found in an individual was four, indicating the presence of at least two loci (assuming all loci are heterozygous). For MHC‐II, the maximum number of alleles was six, suggesting at least three loci. We excluded several individuals with low‐quality MHC sequences from the analyses (Table S1).

MHC‐I blast search indicated that 21 out of the 48 nucleotide sequences were new MHC alleles (GenBank accession numbers: OR578737–OR578757) (Table S2). The most common alleles were ZocaU*2 (60 individuals) and ZocaU*5 (10 individuals). Less than 10 individuals shared the rest of the alleles. For MHC‐II blast, they resulted in 104 haplotypes (GenBank accession numbers: OQ377810–OQ377913). The most common MHC‐II alleles were alleles Zoca1 (85 individuals), Zoca9 (14 individuals), and Zoca16 (13 individuals), all of them new alleles.

### 3.2 Selection and phylogenetic reconstruction

We detected signs of positive selection in both MHC‐I (amino acid positions 12, 48, 51) and MHC‐II (amino acid positions 10, 15, 18, 27, 33, 37, 40, 47, 50, 51, 53, 57, 58). The positions identified in our analysis coincided with those previously observed under positive selection in passerine birds at rates of 9.1% for MHC‐I and 69.2% for MHC‐II (Minias et al., 2018) (Figure S1). And a coincidence rate of 0% for MHC‐I and 35.2% for MHC‐II when compared to human PBR positions (Brown et al., 1993; Saper et al., 1991). Tajima's D neutrality test confirmed a greater number of non‐synonymous versus synonymous substitutions in the PBR compared with non‐PBR regions for both MHC classes (Tables S3 and S4).

### 3.3 Individual parasite infection status

A higher prevalence of *Plasmodium* than *Haemoproteus* was found, with 24% and 12% of birds infected, respectively. The detected parasites corresponded to *Plasmodium* (*Haemamoeba*) *cathemerium* (ZOCAP15), *Plasmodium* (*Novyella*) *homopolare* (BAEBIC02), *Plasmodium* SGS1, *Haemoproteus* (*P*.) *sp1* (ZC1), *Haemoproteus coatneyi, Haemoproteus erythrogravidus, and Haemoproteus* CHLOP01 (Table S1). All these infections have been previously reported (Basto et al., 2006; Cadena, 2015; Cadena‐Ortiz et al., 2019; Doussang et al., 2019; González et al., 2015; Hussing, 2020; Martínez et al., 2016; Marzal et al., 2015), except for the new individuals from Chile to whom PCR, and microscopy was done in this study. Some infections could only be identified at the genus level for some individuals, leaving them as *Haemoproteus* sp. and *Plasmodium* sp.

### 3.4 Associations between MHC allele diversity and latitude

**FIGURE 2:**
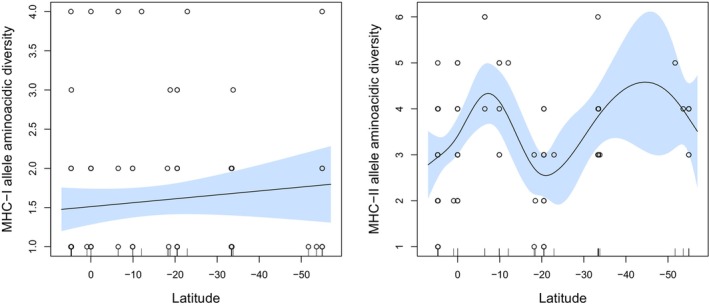
Generalized additive mixed model (GAMM) associations between MHC‐I and MHC‐II amino acidic allele diversity and latitude. 95% confidence intervals are shown.

GAMM model selection for MHC‐I resulted in a best model with only the intercept as the final term, excluding models that incorporated latitude (Table 1, Figure 2). Models including latitude, year, or location were equally plausible based on delta <2, but latitude was not statistically significant in any model. However, in two models, year was statistically significant, indicating a random effect of year on MHC‐I. For MHC‐II, the best GAMM model retained latitude as the final term. MHC‐II amino acid allele diversity was non‐linearly associated with latitude (Table 1, Table S5). Specifically, MHC‐II amino acid allele diversity was low in North Chile (latitude −18° to −20°) and locations near the Equator (0° to 4°), then increased in Peru (−6° to −12°), and further increased from central to the austral region of Chile (−33° to −54°) (Figure 2). Based on delta <2, three other models were also plausible, with latitude always being statistically significant, but not location or year, excluding any random effects of these variables. All models' assumptions were met.

**TABLE 1:** Associations among rufous‐collared sparrow major histocompatibility complex (MHC) amino acidic allele diversity and latitude, year, and location, investigated by generalized additive mixed models (GAMM) and AICc model selection.

		df	logLik	AICc	Delta
MHC‐I	Intercept	0	−121.908	245.861	0
	Latitude + Location	2	−121.685	247.508	1.646
	Latitude	2	−121.685	247.509	1.647
	Latitude + Location + Year	2	−121.504	247.855	1.994
	Latitude + Year	2	−121.504	247.855	1.994
MHC‐II	Latitude	7	−122.012	261.375	0
	Latitude + Location	7	−122.012	261.382	0.006
	Latitude + Year	8	−121.567	262.737	1.361
	Latitude + Year + Location	8	−121.567	262.738	1.362

### 3.5 Associations between infections and latitude/biomes

GAMM models found no association between infection status for either *Plasmodium* or *Haemoproteus* and latitude. The best GAMM model for *Plasmodium* included latitude as an explanatory term, along with several plausible models that included combinations of year and location (Table 2). In the best model and the equally plausible models, the association with latitude were not statistically significant (Table S6, Figure S3). For *Haemoproteus*, the best model showed a marginally significant association with latitude (*p* = .051), with a pattern similar to that observed for MHC‐II, with individuals from north Chile having a low prevalence of infection (Table S6, Figure S3). Another plausible model also included latitude and year as factors.

**TABLE 2:** Associations among rufous‐collared sparrow infection status with latitude, year, and location, investigated by generalized additive mixed models (GAMM) and AICc model selection.

		df	logLik	AICc	Delta
*Plasmodium*	Latitude	4	−27.494	63.549	0
	Latitude + Year	4	−27.494	63.549	0.0001
	Latitude + Location	5	−26.629	65.047	1.498
	Latitude + Year + Location	5	−26.629	65.047	1.498
*Haemoproteus*	Latitude	5	−43.298	98.424	0
	Latitude + Year	5	−43.295	98.433	0.0096

**FIGURE 3:**
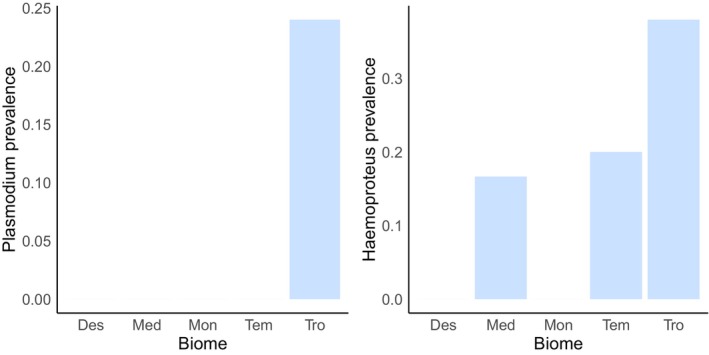
*Plasmodium* and *Haemoproteus* parasite prevalence among biomes. Des = Deserts and Xeric Shrublands, Med = Mediterranean Forests, Mon = Montane Grasslands and Shrublands, Tem = Temperate Broadleaf and Mixed Forests, Woodlands and Scrub, and Tro = Tropical and Subtropical Moist Broadleaf Forests.

Finally, we found a statistically significant association between *Haemoproteus* infection and biome (Fisher's exact test, *p* = .0018). *Haemoproteus* prevalence was higher in the tropical biome compared with the desert biome (post‐hoc Bonferroni test: tropical vs. desert, *n* = 69, adjusted *p* = .007) (Figure 3). There was also a significant association between *Plasmodium* prevalence and biome (Fisher's exact test, *p* = .028); however, this result became non‐significant after the Bonferroni test (post hoc Bonferroni test: tropical vs. desert, *n* = 69, adjusted *p* = .281).

## 4 DISCUSSION

In this study, we explored the relationships between MHC diversity and avian malaria infections (*Plasmodium* and *Haemoproteus*) across a latitudinal gradient in a passerine bird species in South America. While avian MHC is known to respond to a diverse array of antigens, there is compelling evidence linking MHC alleles to avian malaria infections (O'Connor et al., 2019). Based on the latitudinal gradient in biological diversity (LGD) hypothesis, we expected a decrease in MHC allele diversity from the Equator to the austral region of South America, which would correspond to a decrease in the prevalence of *Plasmodium* and *Haemoproteus* parasites at higher latitudes. However, if the prevalence of infection does not follow the trend predicted by the LGD, then MHC diversity should be lower in regions where the prevalence of infection is lower or absent. We found that Individuals from north Chile exhibited the lowest MHC‐II diversity, a finding that aligned with the absence of infections by both parasites. MHC diversity could be in part explained by a low prevalence of avian malaria parasites in the region. This result was supported by the observation that individuals from the deserts and xeric shrublands (intermediate latitude, north Chile) were less infected by *Haemoproteus* parasites compared to individuals from tropical and subtropical biomes.

Although LGD is observed for many plant and animal species, other taxonomic groups, like parasites, have less clear patterns (Clark et al., 2014), even the reverse, as is the case for the *Leucocytooon* parasite, a closely related species (Cuevas et al., 2020; Fecchio et al., 2020; Merino et al., 2008). In rufous‐collared sparrows, in the most complete study to date, the higher prevalence of *Plasmodium* is found in central Chile and in Peru, and for *Haemoproteus* in central and northern Chile (Doussang et al., 2019), and the lowest prevalence for both parasites in Colombia and the austral region of Chile. This trends is supported by a low prevalence for both parasites reported in an Andean community of birds in Colombia (although at elevations ranging from 2100 to 4000 m.a.s.l) (González et al., 2015). Overall, these contrasting results highlight the variability in the patterns of avian malaria prevalence detected in South America. These differences may be better understood by considering the type of biome. North Chile is characterized by deserts and semi‐arid habitats, including the presence of the Atacama Desert, the driest desert on Earth (Darack, 2008). This disrupts the expected linear decreasing latitudinal pattern for the prevalence of avian malaria. Thus, biome could be a factor that better summarizes the effect of latitude, along with other biotic and abiotic factors, on infections (Fecchio et al., 2019). In the case of avian malaria parasites, precipitation and temperature are main factors affecting the life cycle of insect vectors (Doussang et al., 2019; Martínez‐de la Puente et al., 2009). Hence, desert or semi‐arid habitats could limit the occurrence and their activity (Chapa‐Vargas et al., 2020). On the contrary, tropical and subtropical environments may harbour a larger community of insect vectors, which is reflected in the higher prevalence of *Haemoproteus* and greater diversity of MHC‐II compared to desert biomes. However, in locations close to the Equator in South America, a low prevalence of infection have been also reported, possible related to a dilution effect, where high diversity of host birds is associated with a decrease in parasite transmission among hosts of the same species (Moens & Pérez‐Tris, 2016). This could explain the moderate MHC‐II diversity levels observed in our study. However, an intermediate prevalence of *Plasmodium* and *Haemoproteus* (23%–50%, respectively) is also observed in Ecuador (Cadena‐Ortiz et al., 2019), and in our study.

Contrary to our findings, Doussang et al. (2019) reported an elevated prevalence of infection by *Plasmodium* and *Haemoproteus* in rufous‐collared sparrows in northern Chile. In the same region, another study reported *Plasmodium* infecting house sparrows (*Passer domesticus*) but not rufous‐collared sparrows (Martínez et al., 2016), and *Haemoproteus* was observed infecting eared doves (*Zenaida auriculata*). This parasite belongs to the subgenus *Haemoproteus* (different from the subgenus *Parahaemoproteus* that usually infects rufous‐collared sparrows), thus confirming local transmission of *Plasmodium* and at least the *Haemoproteus* subgenus. Differences in results among studies could be due to particularities among sampling localities or other factors, including the sampling period. Although arid, the region presents valleys and oases with water presence where suitable conditions could exist for vectors, favoring local transmission **(**González‐Gómez et al., 2018). One possibility is that the birds concentrate in these oases, generating greater density in those areas and favoring the transmission of the parasite. On the contrary, the prevalence of infection varies with season, so it can confound the actual prevalence if not sampled throughout the year. Birds included in our study were sampled in this region during the austral summer (January) and winter (June–July) in 2012 and 2017, covering a broad period where transmission is expected to occur and in different years. The detection of avian malaria parasites in local birds supports active transmission, but also indicates differential transmission depending on the host species (Clark, 2018; Doussang et al., 2021). Differences in prevalence in bird species from the same area could be related to life‐history traits like nestling period, nest location, vegetation strata, or body mass that increase the exposition to vectors (Quillfeldt et al., 2011). In order to account for this variability, we included year as a random effect in the models, but no effect was observed.

Evidence of low MHC diversity in species living in environments with a low prevalence of parasites has been observed in other birds. For example, the prevalence of blood parasitic infections is low in seabirds, which have been related to the reduced occurrence of vectors in marine environments (Quillfeldt et al., 2011). Moreover, in a latitudinal study ranging from Peru to South Chile evaluating Magellanic and Humboldt penguins, only Humboldt penguins from Peru were infected with *Haemoproteus* compared with southern locations. Precisely, individuals from the infected population exhibited elevated MHC‐I and MHC‐II allele diversity, suggesting a greater diversity associated with the infections (Sallaberry‐Pincheira et al., 2015, 2016). Interestingly, it has been observed that Magellanic penguins translocated to other parts of the world, such as zoos from the northern hemisphere, tend to succumb to infection caused by avian malaria parasites (Hernandez‐Colina et al., 2021). This results supports that parasites' absence or low abundance could result in low MHC allele diversity in rufous‐collared sparrows in this region (Radwan et al., 2010).

Due to the latitudinal amplitude of the work and the small sample size, we could not perform a detailed analysis of the associations between MHC diversity or specific alleles on infections. However, for both types of MHC we have detected positive selection on PBR. In the case of MHC‐I, several alleles were closely related to other alleles previously associated with *Haemoproteus* infections in Perú (Jones et al., 2014, 2015). The most common *Haemoproteus* molecular lineage detected in those studies was *H*. (*P*.) *sp1* (ZC1) (KC480265). This parasite corresponds to the same lineage reported in (Cadena‐Ortiz et al., 2019; Doussang et al., 2019; Merino et al., 2008) and detected in our study in individuals from Ecuador. Its high prevalence in South America is suggested possibly associated with chronic infections of low virulence (Doussang et al., 2019; Merino et al., 2008). This parasite has been related to the morpho species *H. coatneyi* parasite. *H. coatneyi* is closely related to *H. erythrogravidus* (which was also detected in our study), and both share *Z. capensis* as a host. They have a wide distribution and impact various bird species across South America*. H. coatneyi* has a broad geographical distribution, occurring in South, Central, and North America, infecting several species, whereas *H. erythrogravidus* occurs only in South America and is restricted to infecting only two species: the rufous‐collared sparrows and the blue‐winged mountain tanager (*Anisognathus somptuosus*) (F. Thraupidae) (de Oliveira et al., 2020; Valkiūnas, 2005). On the contrary, the other lineage found in our study, CHLOP01, was restricted to *Z. capensis* in Peru (Marzal et al., 2015); however, the virulence of these parasites is unknown. With respect to *Plasmodium* parasites infecting rufous‐collared sparrows we detected *Plasmodium (Haemamoeba) cathemerium* (ZOCAP15), *Plasmodium (Novyella) homopolare* (BAEBIC02), and *Plasmodium relictum* SGS1. In Bosque de Jerusalem in Ecuador, the prevalence of ZOCAP15 was very low, which could mean that it is either rare in the population or actually is very virulent (Cadena‐Ortiz et al., 2019). Interestingly, one individual infected by this lineage had one of the highest parasitemia, suggesting high susceptibility to this lineage. On the contrary, BAEBIC02 has been reported to infect several passerines, mainly species from Emberizidae and Passerellidae families, included rufous‐collared sparrows (Cadena‐Ortiz et al., 2019; Rivero de Aguilar et al., 2018; Walther et al., 2014). *Plasmodium relictum* SGS1 has been recently detected for the first time in Peru and is considered an invasive species for the South American continent. Therefore, it is necessary to continue monitoring these parasites to discern the effects they may have on the host and their role on parasite‐mediated selection on MHC. For example, in Bosque de Jerusalem in Ecuador, the prevalence of both genera was among the highest among all locations, suggesting that individuals cannot avoid infections, instead trying to keep infections at low intensities through their immune system, that is, MHC genes.

Our results generally support variability in MHC diversity on a latitudinal scale related to biome; however, we have also identified other factors that could explain our results. On the one hand, our results support differences both in MHC diversity and infection prevalence in a specific area coinciding with an arid biome, but also a result consistent with the separation between *Zonotrichia* control region‐based molecular lineages observed between northern Chile and Peru. In South America, there is evidence of molecularly distinct clusters based on a mitochondrial control region genes (Lougheed et al., 2013). Thus, the genetic differentiation of these populations could determine the differences observed in the MHC genes. Conversely, differences in MHC diversity may reflect the distinct demographic histories of populations from various regions. Reduced MHC diversity could result from low genetic diversity in bottlenecked or inbred populations (Radwan et al., 2010). Moreover, MHC diversity variation found in our study should reflect adaptation to the local community of parasites, however, selection can be modulated or being stronger toward other pathogens (Llanos‐Soto et al., 2017; Loiseau et al., 2008). Finally, bird movements could be affecting our results' accuracy. The rufous‐collared sparrow is considered a sedentary species in most of the distribution, except for southern Chile, with only one subspecies Z. *c. australis* being a long‐distance migrant from central Chile and Argentina to the Magallanes region. Even for a common species, such as the rufous‐collared sparrow, there is still scarce information about the migratory status of many populations (Medrano et al., 2018). Nevertheless, in a broad latitudinal scale, as is our study, we should have captured latitudinal differences in the MHC related to local parasites.

## AUTHOR CONTRIBUTIONS

**Juan Rivero de Aguilar:** Conceptualization (lead); data curation (lead); formal analysis (lead); funding acquisition (equal); investigation (lead); methodology (equal); project administration (equal); resources (equal); software (equal); supervision (equal); validation (equal); visualization (equal); writing – original draft (equal); writing – review and editing (equal). **Omar Barroso:** Conceptualization (equal); data curation (equal); investigation (equal); methodology (equal); validation (equal); visualization (equal); writing – review and editing (equal). **Elisa Bonaccorso:** Conceptualization (equal); data curation (equal); formal analysis (equal); funding acquisition (equal); investigation (equal); methodology (equal); project administration (equal); resources (equal); software (equal); supervision (equal); validation (equal); visualization (equal); writing – original draft (equal); writing – review and editing (equal). **Hector Cadena:** Data curation (equal); investigation (equal); writing – review and editing (equal). **Lucas Hussing:** Conceptualization (equal); data curation (equal); formal analysis (equal); investigation (equal); methodology (equal); validation (equal); writing – review and editing (equal). **Josefina Jorquera:** Data curation (equal); formal analysis (equal); investigation (equal); methodology (equal); software (equal); supervision (equal); validation (equal); writing – review and editing (equal). **Javier Martinez:** Conceptualization (equal); data curation (equal); funding acquisition (equal); investigation (equal); methodology (equal); project administration (equal); resources (equal); software (equal); supervision (equal); validation (equal); visualization (equal); writing – original draft (equal); writing – review and editing (equal). **Josué Martínez‐de la Puente:** Data curation (equal); investigation (equal); methodology (equal); supervision (equal); validation (equal); visualization (equal); writing – original draft (equal); writing – review and editing (equal). **Alfonso Marzal:** Conceptualization (equal); data curation (equal); formal analysis (equal); investigation (equal); methodology (equal); supervision (equal); validation (equal); visualization (equal); writing – original draft (equal); writing – review and editing (equal). **Fabiola León Miranda:** Conceptualization (equal); data curation (equal); formal analysis (equal); investigation (equal); methodology (equal); software (equal); supervision (equal); validation (equal); visualization (equal); writing – original draft (equal); writing – review and editing (equal). **Santiago Merino:** Conceptualization (equal); funding acquisition (equal); investigation (equal); methodology (equal); project administration (equal); resources (equal); supervision (equal); validation (equal); visualization (equal); writing – original draft (equal); writing – review and editing (equal). **Nubia E. Matta:** Conceptualization (equal); data curation (equal); formal analysis (equal); funding acquisition (equal); investigation (equal); methodology (equal); project administration (equal); resources (equal); software (equal); supervision (equal); validation (equal); visualization (equal); writing – original draft (equal); writing – review and editing (equal). **Marilyn Ramenofsky:** Conceptualization (equal); data curation (equal); funding acquisition (equal); investigation (equal); methodology (equal); project administration (equal); resources (equal); supervision (equal); validation (equal); visualization (equal); writing – original draft (equal); writing – review and editing (equal). **Ricardo Rozzi:** Funding acquisition (equal); project administration (equal); resources (equal); supervision (equal); validation (equal); visualization (equal); writing – original draft (equal); writing – review and editing (equal). **Carlos E. Valeris‐Chacín:** Conceptualization (equal); data curation (equal); investigation (equal); methodology (equal); supervision (equal); validation (equal); writing – original draft (equal); writing – review and editing (equal). **Rodrigo A. Vásquez:** Conceptualization (equal); funding acquisition (equal); investigation (equal); methodology (equal); project administration (equal); resources (equal); supervision (equal); validation (equal); visualization (equal); writing – original draft (equal); writing – review and editing (equal). **Juliana A. Vianna:** Conceptualization (equal); funding acquisition (equal); investigation (equal); methodology (equal); project administration (equal); resources (equal); software (equal); supervision (equal); validation (equal); visualization (equal); writing – original draft (equal); writing – review and editing (equal). **John C. Wingfield:** Conceptualization (equal); data curation (equal); funding acquisition (equal); investigation (equal); methodology (equal); project administration (equal); resources (equal); supervision (equal); validation (equal); visualization (equal); writing – original draft (equal); writing – review and editing (equal).

## FUNDING INFORMATION

This study was funded by FONDECYT‐CONICYT Postdoctorado 3,170,211; CONICYT PIA APOYO CCTE AFB170008; IEB‐FB210006; ANID—Convocatoria Nacional Subvención a la Instalación en la Academia, 2020, PAI77200078; Cape Horn International Center (CHIC), ANID/BASAL FB210018; ANID—Programa Iniciativa Milenio—ICN2021_044 (CGR), ICN2021_002 (BASE), and NCN2021‐050 (LiLi); Universidad San Francisco de Quito (HUBI 12424); JMP was financed by the PID2020‐118205GB‐I00 grant funded by MCIN/AEI/10.13039/501100011033 and by “ERDF A way of making Europe”; AM was funded by line of action LA4 (R + D + I program in the Biodiversity Area financed with the funds of the FEDER Extremadura 2021–2027 Operational Program of the Recovery, Transformation and Resilience Plan) and by “Consejería de Economía e Infraestructura of the Junta de Extremadura” and the European Regional Development Fund, a Way to Make Europe, through the research project IB20089.

## CONFLICT OF INTEREST STATEMENT

The authors have no conflicts of interest to declare.

## Supporting information


Data S1.

## Data Availability

MHC sequencing reads have been deposited at NCBI (GenBank accession numbers: MHC‐I OR578737–OR578757; MHC‐II: OQ377810–OQ377913). Database as well as R code for stats and figures used in this publication are available on Zenodo (10.5281/zenodo.11398506).
