# Supplementary material for: Associations among MHC genes, latitude, and avian malaria infections in the rufous‐collared sparrow (Zonotrichia capensis)
Source: Ecol Evol. 2024 Jul 17;14(7):e11634. doi: 10.1002/ece3.11634 (PMC11255377; doi:10.1002/ece3.11634)
Supplement: Supplementary file 1 — Data S1. [file ECE3-14-e11634-s001.docx]

**Supporting Information for online publication**

**Associations among MHC genes, latitude, and haemosporidian infections in the rufous-collared sparrow (*Zonotrichia capensis*)**

Juan Rivero de Aguilar, Omar Barroso, Elisa Bonaccorso, Hector Cadena, Lucas Hussing, Josefina Jorquera, Javier Martinez, Josué Martínez-de la Puente, Alfonso Marzal, Fabiola Miranda, Santiago Merino, Nubia E. Matta, Marilyn Ramenofsky, Ricardo Rozzi, Carlos E. Valeris-Chacín, Rodrigo Vásquez, Juliana A. Vianna , John C. Wingfield

Table 1. MHC-I and -II alleles found in each country and *Plasmodium* and *Haemoproteus* infections. M = examined by microscopy. PCR = examined by PCR. Biome: Tr = Tropical and Subtropical Moist Broadleaf Forests, Te = Temperate Broadleaf and Mixed Forests, Mo = Montane Grasslands & Shrublands, Me = Mediterranean Forests, Woodlands and Scrub and De = Deserts and Xeric Shrublands.

| Country | Locality | Biome | N | Individual | MHC-I nucleotide div | MHC-I amino div | MHC-I allele (accession number) | MHC-II nucleotide div | MHC-II amino div | MHC allele (accession number) | Plas | Hae |
| --- | --- | --- | --- | --- | --- | --- | --- | --- | --- | --- | --- | --- |
| Colombia | Aldana | Tr | 1 | AN07 | 1 | 1 | ZocaU*2 | 2 | 2 | Zoca1  Zoca65 | - (M) | - (M) |
|  | Parque Nacional Natural Los Nevados, vereda El Bosque | Mo | 3 | OT1108 | 1 | 1 | ZocaU*2 | 3 | 3 | Zoca1  Zoca36  Zoca63 | - (M) | - (M) |
|  |  | Mo |  | OT906 | 1 | 1 | ZocaU*15 | 3 | 3 | Zoca1  Zoca9  Zoca25 | - (M) | - (M) |
|  |  | Mo |  | OT910 | 4 | 4 | ZocaU*2  ZocaU*104  ZocaU*105  ZocaU*106 | 2 | 2 | Zoca1  Zoca26 | - (M) | - (M) |
|  | Santuario de Fauna y Flora Otún Quimbaya | Tr | 2 | OT1203 | 1 | 1 | ZocaU*2 | 2 | 2 | Zoca1  Zoca58 | - (M) | - (M) |
|  |  | Tr |  | OT1219 | 2 | 1 (Zoca2 y New1 =proteina) | ZocaU*2  ZocaU*101 | 3 | 3 | Zoca1  Zoca23  Zoca24 | - (M) | - (M) |
|  | Parque Regional Natural Ucumarí | Tr | 6 | OT703 | 2 | 2 | ZocaU*2  ZocaU*3 | 2 | 2 | Zoca1  Zoca9 | - (M) | - (M) |
|  |  | Tr |  | OT941 | 1 | 1 | ZocaU*101 | 4 | 4 | Zoca1  Zoca9  Zoca26  Zoca27 | - (M) | - (M) |
|  |  | Tr |  | OT957 | 1 | 1 | ZocaU*9 | 3 | 3 | Zoca1  Zoca9  Zoca26 | + (M/PCR)  *P. homopolare* | - (M) |
|  |  | Tr |  | OT960 | 1 | 1 | ZocaU*2 | 2 | 2 | Zoca1  Zoca27 | - (M) | - (M) |
|  |  | Tr |  | OT963 | 2 | 2 | ZocaU*2  ZocaU*9 | 1 | 1 | Zoca1 | - (M) | - (M) |
|  |  | Tr |  | OT969 | 1 | 1 | ZocaU*9 | 4 | 4 | Zoca1  Zoca24  Zoca59  Zoca60 | - (M) | + (M/PCR)  *H. coatneyi* |
|  | Parque Nacional Natural Los Nevados, Estación El Cedral | Tr | 3 | OT1161 | 1 | 1 | ZocaU*2 | 4 | 4 | Zoca1  Zoca26  Zoca36  Zoca63 | + (M/PCR)  *P. homopolare* | - (M) |
|  |  | Tr |  | OT1173 | 2 | 2 | ZocaU*2  ZocaU*7 | 5 | 5 | Zoca1  Zoca26  Zoca38  Zoca48  Zoca64 | - (M) | + (M/PCR)  *Haemoproteus* sp. |
|  |  | Tr |  | OT998 | 1 | 1 | ZocaU*24 | 3 | 3 | Zoca1  Zoca9  Zoca24 | - (M) | + (M/PCR)  *H. coatneyi* |
|  | Parque Nacional Natural Chingaza, Estación Palacio | Tr | 1 | PA239 | 1 | 1 | ZocaU*103 | 4 | 4 | Zoca1  Zoca9  Zoca28  Zoca29 | - (M) | - (M) |
|  | Campus Universidad Nacional de Colombia | Tr | 5 | UN290 | 2 | 1 ( ZocaU*2  ZocaU*15=proteina) | ZocaU*2  ZocaU*15 | 2 | 2 | Zoca1  Zoca9 | - (M) | + (M/PCR)  *Haemoproteus* sp. |
|  |  | Tr |  | UN301 | 1 | 1 | ZocaU*103 | 4 | 4 | Zoca1  Zoca28  Zoca48  Zoca61 | - (M) | - (M) |
|  |  | Tr |  | UN307 | 4 | 3 ( ZocaU*2  ZocaU*52  =prot) | ZocaU*2  ZocaU*52  ZocaU*120 | 1 | 1 | Zoca30 | - (M) | - (M) |
|  |  | Tr |  | UN315 | NGS low quality | - | - | 5 | 4 | Zoca31  Zoca32  Zoca33  Zoca34  Zoca35 | - (M) | - (M) |
|  |  | Tr |  | UN352 | 1 | 1 | ZocaU*69 | 4 | 4 | Zoca1  Zoca48  Zoca61  Zoca62 | - (M) | + (M/PCR)  *H. erythrogravidus* |
| Ecuador | Bosque Protector Jerusalem | Tr | 21 | HFC 003 | 2 | 1( ZocaU*2  ZocaU*52  =prot) | ZocaU*2  ZocaU*52 | 5 | 5 | Zoca1  Zoca12  Zoca36  Zoca37  Zoca38 | - (M/PCR) | + (M/PCR)  *H. erythrogravidus* and  *H. (P.) sp1* (ZC1) (KC480265) |
|  |  | Tr |  | HFC 061 | 1 | 1 | ZocaU*2 | 5 | 5 | Zoca1  Zoca38  Zoca43  Zoca48  Zoca57 | + (M/PCR)  *P. (N.) homopolare* (BAEBIC02) (KF537287) | - (M/PCR) |
|  |  | Tr |  | HFC 078 | 1 | 1 | ZocaU*2 | 3 | 3 | Zoca1  Zoca11  Zoca39 | + (M/PCR)  *P. (N.) homopolare* (BAEBIC02)  (KF537287) | - (M/PCR) |
|  |  | Tr |  | HFC 079 | 1 | 1 | ZocaU*2 | 2 | 2 | Zoca1  Zoca40 | *+* (M/PCR)  *P. (N.) homopolare* (BAEBIC02)  (KF537287) | *+* (M/PCR)  *H (P.) sp1* (ZC1) (KC480265) |
|  |  | Tr |  | HFC 085 | 1 | 1 | ZocaU*102 | 3 | 3 | Zoca1  Zoca41  Zoca42 | + (M/PCR)  *P. (N.) homopolare* (BAEBIC02)  (KF537287) | - (M/PCR) |
|  |  | Tr |  | HFC 100 | 1 | 1 | ZocaU*2 | 2 | 2 | Zoca1  Zoca43 | -(M/PCR) | -(M/PCR) |
|  |  | Tr |  | HFC 105 | 1 | 1 | ZocaU*2 | 4 | 4 | Zoca1  Zoca20  Zoca43  Zoca44 | -(M/PCR) | -(M/PCR) |
|  |  | Tr |  | HFC 157 | 1 | 1 | ZocaU*2 | 2 | 2 | Zoca1  Zoca45 | -(M/PCR) | + (M/PCR)  *H. erythrogravidus* and  *H. (P.) sp1* (ZC1) (KC480265) |
|  |  | Tr |  | HFC 176 | 1 | 1 | ZocaU*2 | 4 | 4 | Zoca1  Zoca36  Zoca46  Zoca47 | -(M/PCR) | +(M/PCR)  *H. (P.) sp1* (ZC1) (KC480265) |
|  |  | Tr |  | HFC 185 | 3 | 2 ( ZocaU*2  ZocaU*4  =prot) | ZocaU*2  ZocaU*4  ZocaU*49 | 3 | 3 | Zoca1  Zoca43  Zoca48 | -(M/PCR) | -(M/PCR) |
|  |  | Tr |  | HFC 187 | 1 | 1 | ZocaU*2 | 3 | 3 | Zoca1  Zoca36  Zoca38 | -(M/PCR) | -(M/PCR) |
|  |  | Tr |  | HFC 190 | 2 | 1 ( ZocaU*2  ZocaU*4=prot) | ZocaU*2  ZocaU*4 | 3 | 3 | Zoca1  Zoca49  Zoca50 | -(M/PCR) | +(M/PCR)  *H. (P.) sp1* (ZC1) (KC480265) |
|  |  | Tr |  | HFC 204 | 2 | 2 | ZocaU*2  ZocaU*110 | 4 | 4 | Zoca1  Zoca43  Zoca48  Zoca57 | + (M/PCR)  *P. (H.) cathemerium* (ZOCAP15) (MK077679) | +(M/PCR)  *H. (P.) sp1* (ZC1) (KC480265) |
|  |  | Tr |  | HFC 269 | 2 | 2 | ZocaU*52  ZocaU*109 | 2 | 2 | Zoca1  Zoca47 | -(M/PCR) | +(M/PCR)  *H. (P.) sp1* (ZC1) (KC480265) |
|  |  | Tr |  | HFC 327 | 2 | 2 | ZocaU*2  ZocaU*109 | 3 | 3 | Zoca1  Zoca27  Zoca43 | +(M/PCR)  *P. (N.) homopolare* (BAEBIC02)  (KF537287) | -(M/PCR) |
|  |  | Tr |  | HFC 337 | 1 | 1 | ZocaU*102 | 4 | 4 | Zoca1  Zoca36  Zoca47  Zoca51 | -(M/PCR) | -(M/PCR) |
|  |  | Tr |  | HFC 352 | 1 | 1 | ZocaU*4 | 4 | 4 | Zoca1  Zoca12  Zoca23  Zoca52 | -(M/PCR) | +(M/PCR)  *H. (P.) sp1* (ZC1) (KC480265) |
|  |  | Tr |  | HFC 406 | 2 | 2 | ZocaU*15  ZocaU*32 | 5 | 5 | Zoca1  Zoca17  Zoca38  Zoca53  Zoca54 | -(M/PCR) | -(M/PCR) |
|  |  | Tr |  | HFC 481 | 2 | 2 | ZocaU*2  ZocaU*110 | 3 | 3 | Zoca1  Zoca26  Zoca56 | -(M/PCR) | +(M/PCR)  *H. (P.) sp1* (ZC1) (KC480265) |
|  |  | Tr |  | HFC 629 | 1 | 1 | ZocaU*2 | 3 | 3 | Zoca1  Zoca23  Zoca55 | +(M/PCR)  *P. (N.) homopolare* (BAEBIC02)  (KF537287) | -(M/PCR) |
|  |  | Tr |  | HFC 697 | 4 | 4 | ZocaU*2  ZocaU*104  ZocaU*105  ZocaU*106 | 3 | 3 | Zoca1  Zoca36  Zoca43 | -(M/PCR) | +(M/PCR)  *H. (P.) sp1* (ZC1) (KC480265) |
|  | Tarapoto aeropuerto | Tr | 3 | P16127 | 4 | 4 | ZocaU*2  ZocaU*5  ZocaU*13  ZocaU*121 | 6 | 6 | Zoca1  Zoca2  Zoca3  Zoca4  Zoca5  Zoca6 | - (PCR) | - (PCR) |
|  |  | Tr |  | P16130 | 2 | 2 | ZocaU*24  ZocaU*57 | 6 | 6 | Zoca1  Zoca2  Zoca7  Zoca8  Zoca9  Zoca10 | -(PCR) | -(PCR) |
|  |  | Tr |  | P16132 | 1 | 1 | ZocaU*13 | 4 | 4 | Zoca1  Zoca7  Zoca11  Zoca12 | -(PCR) | -(PCR) |
| Perú | Huanuco, Las Pampas | Tr | 7 | P12172 | 1 | 1 | ZocaU*73 | 3 | 3 | Zoca1  Zoca9  Zoca63 | -(PCR) | + (PCR)  *Haemoproteus*  CHLOP01 |
|  |  | Tr |  | P12205 | 4 | 2 | ZocaU*15  ZocaU*69  ZocaU*20  ZocaU*111 | 5 | 5 | Zoca1  Zoca9  Zoca17  Zoca71  Zoca72 | +(PCR)  *Plasmodium*  SGS1 | -(PCR) |
|  |  | Tr |  | P12228 | 2 | 2 | ZocaU*2  ZocaU*32 | 3 | 3 | Zoca1  Zoca9  Zoca73 | +(PCR)  *P. (N.) homopolare* (BAEBIC02)  (KF537287) | -(PCR) |
|  |  | Tr |  | P12232 | 1 | 1 | ZocaU*2 | NGS low quality | - | - | -(PCR) | + (PCR)  *Haemoproteus*  CHLOP01 |
|  |  | Tr |  | P12237 | 1 | 1 | ZocaU*2 | NGS low quality | - | - | -(PCR) | -(PCR) |
|  |  | Tr |  | P12239 | 2 | 1 | ZocaU*2 | 5 | 5 | Zoca1  Zoca9  Zoca16  Zoca74  Zoca75 | -(PCR) | + (PCR)  *Haemoproteus*  CHLOP01 |
|  |  | Tr |  | P12242 | 1 | 1 | ZocaU*7 | 4 | 4 | Zoca1  Zoca52  Zoca63  Zoca76 | +(PCR)  *P. (N.) homopolare* (BAEBIC02)  (KF537287) | -(PCR) |
|  | UNFV, Lima | De | 1 | P17138 | 4 | 4 | ZocaU*1  ZocaU*3  ZocaU*28  ZocaU*107 | 5 | 5 | Zoca1  Zoca12  Zoca13  Zoca14  Zoca15 | -(PCR) | -(PCR) |
| Chile | Putre | Mo | 2 | RV1790 | 2 | 2 | ZocaU*5  ZocaU*45 | 3 | 3 | Zoca1  Zoca94  Zoca95 | -(M/PCR) | -(M/PCR) |
|  |  |  |  | RV1791 |  |  |  | 1 | 1 | Zoca1 | -(M/PCR) | -(M/PCR) |
|  | Arica | De | 1 | RV1787 | 2 | 1 | ZocaU*2  ZocaU*4 | 2 | 2 | Zoca1  Zoca16 | -(M/PCR) | -(M/PCR) |
|  | Codpa | De | 1 | RV1799 | 3 | 3 | ZocaU*1ZocaU*5ZocaU*80 | NGS low quality | - | - | -(M/PCR) | -(M/PCR) |
|  | El Yeso, Región Metropolitana, Chile | Mo | 1 | RV1786 | 3 | 3 | ZocaU*2  ZocaU*3  ZocaU*113 | 3 | 3 | Zoca1  Zoca88  Zoca89 | -(M) | -(M) |
|  | Iquique-Pica | De | 15 | 7 | 1 | 1 | ZocaU*2 | 3 | 3 | Zoca1  Zoca16  Zoca17 | -(M/PCR) | -(M/PCR ) |
|  |  | De |  | 24 | 2 | 2 | ZocaU*2  ZocaU*5 | 3 | 3 | Zoca1  Zoca16  Zoca18 | -(M/PCR) | -(M/PCR) |
|  |  | De |  | 34 | 1 | 1 | ZocaU*2 |  |  |  | -(M/PCR) | -(M/PCR) |
|  |  | De |  | 54 | 1 | 1 | ZocaU*2 | 2 | 2 | Zoca1  Zoca16 | -(M/PCR) | -(M/PCR) |
|  |  | De |  | 97 | 1 | 1 | ZocaU*52 | 3 | 3 | Zoca1  Zoca16  Zoca19 | -(M/PCR) | -(M/PCR) |
|  |  | De |  | 123 | 1 | 1 | ZocaU*2 | 4 | 4 | Zoca1  Zoca16  Zoca17  Zoca20 | -(M/PCR) | -(M/PCR) |
|  |  | De |  | 126 | 3 | 3 | ZocaU*2  ZocaU*5  ZocaU*118 | 2 | 2 | Zoca1  Zoca16 | -(M/PCR) | -(M/PCR) |
|  |  | De |  | 130 | 2 | 2 | ZocaU*2  ZocaU*5 | 1 | 1 | Zoca1 | -(M/PCR) | -(M/PCR) |
|  |  | De |  | 133 | 2 | 2 | ZocaU*2  ZocaU*13 | 1 | 1 | Zoca1 | -(M/PCR) | -(M/PCR) |
|  |  | De |  | 142 | 3 | 3 | ZocaU*5  ZocaU*17  ZocaU*37 | 3 | 3 | Zoca1  Zoca16  Zoca17 | -(M/PCR) | -(M/PCR) |
|  |  | De |  | 162 | 3 | 2 (Zoca4 y 2 =proteina) | ZocaU*2  ZocaU*4  ZocaU*45 | 2 | 2 | Zoca1  Zoca16 | -(M/PCR) | -(M/PCR) |
|  |  | De |  | 168 | 2 | 2 | ZocaU*5  ZocaU*36 | 4 | 4 | Zoca1  Zoca18  Zoca21  Zoca22 | -(M/PCR) | -(M/PCR) |
|  |  | De |  | 170 | 1 | 1 | ZocaU*2 | 2 | 2 | Zoca1  Zoca17 | -(M/PCR) | -(M/PCR) |
|  |  | De |  | 176 | 1 | 1 | ZocaU*2 | 3 | 3 | Zoca1  Zoca16  Zoca21 | -(M/PCR) | -(M/PCR) |
|  |  | De |  | 188 | 1 | 1 | ZocaU*2 | 1 | 1 | Zoca1 | -(M/PCR) | -(M/PCR) |
|  | San Pedro de Atacama | Mo | 1 | RV1892 | 4 | 2 | ZocaU*4  ZocaU*26  ZocaU*38  ZocaU*97 | 3 | 3 | Zoca1  Zoca14  Zoca68 | - (M/PCR) | -(M/PCR) |
|  | Farellones | Mo | 2 | RV1958 | 2 | 2 | ZocaU*2  ZocaU*5 | 3 | 2 | Zoca1  Zoca52  Zoca87 | -(M/PCR) | -(M/PCR) |
|  |  | Mo |  | RV1959 | 1 | 1 | ZocaU*20 | 6 | 6 | Zoca1  Zoca3  Zoca9  Zoca87  Zoca99  Zoca100 | -(M/PCR) | -(M/PCR) |
|  | Parque Natural San Carlos de Apoquindo, Región Metropolitana | Te | 2 | RV1781 | 2 | 1 | ZocaU*2  ZocaU*4 | NGS low quality | - | - | -(M/PCR) | +(M/PCR)  *H. coatneyi* |
|  |  | Te |  | RV1782 | 1 | 1 | ZocaU*2 | 6 | 4 | Zoca1  Zoca52  Zoca87  Zoca91  Zoca92  Zoca93 | -(M/PCR) | -(M/PCR) |
|  | Isla Navarino | Te |  | RV1080 | 4 | 4 | ZocaU*2  ZocaU*9  ZocaU*20  ZocaU*112 | 4 | 4 | Zoca1  Zoca77  Zoca78  Zoca79 | -(M) | -(M) |
|  |  | Te |  | RV1081 | 2 | 1 | ZocaU*2  ZocaU*15 | 3 | 3 | Zoca1  Zoca80  Zoca81 | -(M/PCR) | -(M/PCR) |
|  |  | Te | 6 | RV1100 | 2 | 2 | ZocaU*2  ZocaU*110 | 4 | 4 | Zoca1  Zoca7  Zoca38  Zoca82 | -(M/PCR) | -(M/PCR) |
|  |  | Te |  | RV1932 | 2 | 1 | ZocaU*2  ZocaU*15 | 4 | 4 | Zoca1  Zoca7  Zoca38  Zoca96 | -(M/PCR) | -(M/PCR) |
|  |  | Te |  | RV1934 | 3 | 2 ( ZocaU*New10  ZocaU*20  =prot) | ZocaU*20  ZocaU*110  ZocaU*116 | 4 | 4 | Zoca1  Zoca38  Zoca77  Zoca97 | -(M/PCR) | -(M/PCR) |
|  |  | Te |  | RV1935 | 1 | 1 | ZocaU*2 | 4 | 3 | Zoca1  Zoca58  Zoca101  Zoca102 | -(M) | -(M) |
|  | Parque Estrecho de Magallanes | Te |  | RV884 | 1 | 1 | ZocaU*2 | 4 | 4 | Zoca1  Zoca85  Zoca86  Zoca87 | -(M/PCR) | -(M/PCR) |
|  | Pica | De | 1 | RV1885 | 1 | 1 | ZocaU*52 | 4 | 4 | Zoca1  Zoca14  Zoca22  Zoca90 | -(M/PCR) | -(M/PCR) |
|  | Puerto Natales | Te | 1 | RV888 | 2 | 1 | ZocaU*114  ZocaU*115 | 5 | 5 | Zoca1  Zoca16  Zoca17  Zoca103  Zoca104 | -(M/PCR) | +  *H. coatneyi* |
|  | Quebrada de la Plata, Región Metropolitana | Me | 5 | RV1777 | 4 | 2 | ZocaU*2  ZocaU*4  ZocaU*119  ZocaU*25 | 4 | 4 | Zoca1  Zoca38  Zoca83  Zoca84 | -(M/PCR) | -(M/PCR) |
|  |  | Me |  | RV1951 | 1 | 1 | ZocaU*2 | 4 | 4 | Zoca1  Zoca9  Zoca82  Zoca98 | -(M/PCR) | -(M/PCR) |
|  |  | Me |  | RV1954 | 2 | 2 | ZocaU*2  ZocaU*117 | 4 | 3 | Zoca1  Zoca16  Zoca19  Zoca90 | -(M/PCR) | -(M/PCR) |
|  |  | Me |  | RV1966 | 1 | 1 | ZocaU*45 | 4 | 4 | Zoca1  Zoca68  Zoca69  Zoca70 | -(M/PCR) | -(M/PCR) |
|  |  | Me |  | RV1972 | 2 | 2 | ZocaU*2  ZocaU*5 | 4 | 4 | Zoca1  Zoca17  Zoca66  Zoca67 | -(M/PCR) | +(M/PCR)  *H. erythogravidus* |

Table 2. Percentage similarity among new MHC-I and -II alleles and other passerine birds from GenBank.

|  | New MHC allele (GenBank) | Percentage identity % | Bird species | MHC Allele (GenBank) |
| --- | --- | --- | --- | --- |
| MHCI | ZocaU*101 (OR578737) | 99.53 | *Zonotrichia capensis* | ZocaU*2 (KF433978) |
|  | ZocaU*102 (OR578738) | 93.53 | *Zonotrichia capensis* | ZocaU*2 (KF433978) |
|  | ZocaU*103 (OR578739) | 93.63 | *Zonotrichia albicolis* | LOC102067888 (XM_014275373.2) PREDICTED |
|  | ZocaU*104 (OR578740) | 98.13 | *Zonotrichia capensis* | ZocaU*45 (KF434021) |
|  | ZocaU*105 (OR578741) | 99.07 | *Zonotrichia capensis* | ZocaU*2 (KF433978) |
|  | ZocaU*106 (OR578742) | 99.0 | *Zonotrichia capensis* | ZocaU*4 (KF433980) |
|  | ZocaU*107 (OR578743) | 99.7 | *Zonotrichia capensis* | ZocaU*60 (KF434036) |
|  | ZocaU*108 (OR578744) | 97.2 | *Zonotrichia capensis* | ZocaU*40 (KF434016) |
|  | ZocaU*109 (OR578745) | 99.53 | *Zonotrichia capensis* | ZocaU*2 (KF433978) |
|  | ZocaU*110 (OR578746) | 99.53 | *Zonotrichia capensis* | ZocaU*20 (KF433996) |
|  | ZocaU*111 (OR578747) | 99.53 | *Zonotrichia capensis* | ZocaU*20 (KF433996) |
|  | ZocaU*112 (OR578748) | 99.53 | *Zonotrichia capensis* | ZocaU*20 (KF433996) |
|  | ZocaU*113 (OR578749) | 99.07 | *Zonotrichia capensis* | ZocaU*42 (KF434018) |
|  | ZocaU*114 (OR578750) | 93.02 | *Zonotrichia capensis* | ZocaU*65 (KF434041) |
|  | ZocaU*115 (OR578751) | 93.02 | *Zonotrichia capensis* | ZocaU*65 (KF434041) |
|  | ZocaU*116 (OR578752) | 97.12 | *Zonotrichia capensis* | ZocaU*78 (KF434054) |
|  | ZocaU*117 (OR578753) | 99.53 | *Zonotrichia capensis* | ZocaU*2 (KF433978) |
|  | ZocaU*118 (OR578754) | 99.07 | *Zonotrichia capensis* | ZocaU*30 (KF434006) |
|  | ZocaU*119 (OR578755) | 99.53 | *Zonotrichia capensis* | ZocaU*25 (KF434001) |
|  | ZocaU*120 (OR578756) | 92.2 | *Cercotrichas podobe* | (MF478253) |
|  | ZocaU*121 (OR578757) | 99.53 | *Zonotrichia capensis* | ZocaU*4 (KF433980) |
|  |  |  |  |  |
| MHCII | Zoca01 (OQ377810) | 98.41 | *Melospiza melodia* | DAB*518 (MH671095) |
|  | Zoca02 (OQ377811) | 94.71 | *Ramphocelus carbo* | C28E2 (AB531656) |
|  | Zoca03 (OQ377812) | 97.88 | *Melospiza melodia* | DAB*518 (MH671095) |
|  | Zoca04 (OQ377813) | 92.59 | *Melospiza melodia* | DAB*232 (KX375269) |
|  | Zoca05 (OQ377814) | 90.48 | *Melospiza melodia* | DAB*374 (MH670991) |
|  | Zoca06 (OQ377815) | 90.53 | *Emberiza jankowskii* | DAB*24 (KT751222) |
|  | Zoca07 (OQ377816) | 97.88 | *Melospiza melodia* | DAB*518 (MH671095) |
|  | Zoca08 (OQ377817) | 95.79 | *Melospiza melodia* | DAB*373 (MH670990) |
|  | Zoca09 (OQ377818) | 91.01 | *Melospiza melodia* | DAB*374 (MH670991) |
|  | Zoca10 (OQ377819) | 92.06 | *Melospiza melodia* | DAB*374 (MH670991) |
|  | Zoca11 (OQ377820) | 94.09 | *Acanthis flammea* | DAB*17 (MW740899) |
|  | Zoca12 (OQ377821) | 92.59 | *Camarhynchus pauper* | D10BMG3 (AB531603) |
|  | Zoca13 (OQ377822) | 91.05 | *Melospiza melodia* | DAB*148 (KX264104) |
|  | Zoca14 (OQ377823) | 92.55 | *Melospiza melodia* | DAB*574 (MK504139) |
|  | Zoca15 (OQ377824) | 97.88 | *Melospiza melodia* | DAB*518 (MH671095) |
|  | Zoca16 (OQ377825) | 92.63 | *Melospiza melodia* | DAB*475 (MH671064) |
|  | Zoca17 (OQ377826) | 98.40 | *Melospiza melodia* | DAB*518 (MH671095) |
|  | Zoca18 (OQ377827) | 90.48 | *Melospiza melodia* | DAB*320 (MF197788) |
|  | Zoca19 (OQ377828) | 95.24 | *Melospiza melodia* | DAB*515 (MH671092) |
|  | Zoca20 (OQ377829) | 95.72 | *Emberiza calandra* | DAB*26 (MW741162) |
|  | Zoca21 (OQ377830) | 97.88 | *Melospiza melodia* | DAB*518 (MH671095) |
|  | Zoca22 (OQ377831) | 97.88 | *Melospiza melodia* | DAB*518 (MH671095) |
|  | Zoca23 (OQ377832) | 93.65 | *Geothlypis trichas* | DAB*914 (JX214984) |
|  | Zoca24 (OQ377833) | 92.11 | *Corvus brachyrhynchos* | CobrIIB22 (KP888339) |
|  | Zoca25 (OQ377834) | 92.59 | *Melospiza melodia* | DAB*104 (KX264060) |
|  | Zoca26 (OQ377835) | 92.06 | *Melospiza melodia* | DAB*22 (KX263978) |
|  | Zoca27 (OQ377836) | 94.15 | *Geothlypis trichas* | DAB*1203 (MH538490) |
|  | Zoca28 (OQ377837) | 94.18 | *Melospiza melodia* | DAB*387 (MH671004) |
|  | Zoca29 (OQ377838) | 90.48 | *Melospiza melodia* | DAB*374 (MH670991) |
|  | Zoca30 (OQ377839) | 89.42 | *Turdus merula* | Tume1-03 (HQ539462) |
|  | Zoca31 (OQ377840) | 98.40 | *Turdus migratorius* | Tumi2-13 (HQ539426) |
|  | Zoca32 (OQ377841) | 97.34 | *Turdus migratorius* | Tumi2-13 (HQ539426) |
|  | Zoca33 (OQ377842) | 89.56 | *Turdus migratorius* | Tumi5-01 (HQ539450) |
|  | Zoca34 (OQ377843) | 97.87 | *Turdus migratorius* | Tumi2-13 (HQ539426) |
|  | Zoca35 (OQ377844) | 97.34 | *Turdus migratorius* | Tumi2-13 (HQ539426) |
|  | Zoca36 (OQ377845) | 94.09 | *Emberiza calandra* | DAB*26 (MW741162) |
|  | Zoca37 (OQ377846) | 92.06 | *Melospiza melodia* | DAB*298 (KX375334) |
|  | Zoca38 (OQ377847) | 92.71 | *Melospiza melodia* | DAB*511 (MF197840) |
|  | Zoca39 (OQ377848) | 89.30 | *Emberiza calandra* | DAB*23 (MW741159) |
|  | Zoca40 (OQ377849) | 91.01 | *Geothlypis trichas* | DAB*317 (JX214414) |
|  | Zoca41 (OQ377850) | 89.78 | *Chloris chloris* | Chch-DAB*1 (MW740977) |
|  | Zoca42 (OQ377851) | 93.65 | *Melospiza melodia* | DAB*494 (MF197837) |
|  | Zoca43 (OQ377852) | 92.59 | *Melospiza melodia* | DAB*22 (KX263978) |
|  | Zoca44 (OQ377853) | 93.65 | *Emberiza jankowskii* | Emja-DAB*19 (KT751217) |
|  | Zoca45 (OQ377854) | 93.12 | *Geothlypis trichas* | DAB*792 (JX214865) PREDICTED |
|  | Zoca46 (OQ377855) | 93.55 | *Acanthis flammea* | Acfl-DAB*17 (MW740899.1) |
|  | Zoca47 (OQ377856) | 92.59 | *Geothlypis trichas* | DAB*937 (JX215007) PREDICTED |
|  | Zoca48 (OQ377857) | 93.16 | *Melospiza melodia* | DAB*574 (MK504139) |
|  | Zoca49 (OQ377858) | 96.79 | *Emberiza calandra* | DAB*26 (MW741162) |
|  | Zoca50 (OQ377859) | 94.62 | *Emberiza calandra* | DAB*35 (MW741171) |
|  | Zoca51 (OQ377860) | 93.12 | *Melospiza melodia* | Sosp-DAB*415 (MH671017) PREDICTED |
|  | Zoca52 (OQ377861) | 99.47 | *Zonotrichia capensis* | Clone C16E1 (AB531666) |
|  | Zoca53 (OQ377862) | 92.59 | *Melospiza melodia* | DAB*120 (KX264076) |
|  | Zoca54 (OQ377863) | 92.11 | *Melospiza melodia* | DAB*475 (MH671064) |
|  | Zoca55 (OQ377864) | 92.02 | *Melospiza melodia* | DAB*119 (MH671064)/Non-functional |
|  | Zoca56 (OQ377865) | 97.88 | *Melospiza melodia* | DAB*518 (MH671095) |
|  | Zoca57 (OQ377866) | 92.59 | *Geothlypis trichas* | DAB*253 (JX214352) |
|  | Zoca58 (OQ377867) | 94.71 | *Melospiza melodia* | DAB*41 (KX263997) |
|  | Zoca59 (OQ377868) | 99.47 | *Zonotrichia capensis* | C16E1 (AB531665) |
|  | Zoca60 (OQ377869) | 91.53 | *Melospiza melodia* | DAB*374 (MH670991) |
|  | Zoca61 (OQ377870) | 92.06 | *Geothlypis trichas* | Getr-DAB*1010 (JX215079) |
|  | Zoca62 (OQ377871) | 89.47 | *Ramphocelus carbo* | C28E2 (AB531656) |
|  | Zoca63 (OQ377872) | 100 | *Zonotrichia capensis* | C16E3 (AB531638) |
|  | Zoca64 (OQ377873) | 94.62 | *Emberiza calandra* | DAB*26 (MW741162) |
|  | Zoca65 (OQ377874) | 88.89 | *Certhidea olivacea* | D4FMG2 (AB531515) |
|  | Zoca66 (OQ377875) | 91.53 | *Melospiza melodia* | DAB*467 (MH671058) |
|  | Zoca67 (OQ377876) | 97.86 | *Melospiza melodia* | DAB*518 (MH671095) |
|  | Zoca68 (OQ377877) | 97.33 | *Melospiza melodia* | DAB*518 (MH671095) |
|  | Zoca69 (OQ377878) | 97.88 | *Melospiza melodia* | DAB*518 (MH671095) |
|  | Zoca70 (OQ377879) | 97.86 | *Melospiza melodia* | DAB*518 (MH671095) |
|  | Zoca71 (OQ377880) | 93.01 | *Coccothraustes coccothraustes* | Coco-DAB*18 (MW741008.1) |
|  | Zoca72 (OQ377881) | 92.59 | *Emberiza jankowskii* | Emja-DAB*17 (KT751215) |
|  | Zoca73 (OQ377882) | 93.12 | *Geothlypis trichas* | Getr-DAB*792 (JX214865) |
|  | Zoca74 (OQ377883) | 92.63 | *Melospiza melodia* | Sosp-DAB*152 (KX264108) |
|  | Zoca75 (OQ377884) | 92.11 | *Geothlypis trichas* | Getr-DAB*1111 (KP735876) |
|  | Zoca76 (OQ377885) | 94.71 | *Geothlypis trichas* | Getr-DAB*937 (JX215007) |
|  | Zoca77 (OQ377886) | 95.83 | *Melospiza melodia* | DAB*511 (MF197840) |
|  | Zoca78 (OQ377887) | 92.59 | *Melospiza melodia* | DAB*22 (KX263978) |
|  | Zoca79 (OQ377888) | 91.53 | *Melospiza melodia* | DAB*467 (MH671058) |
|  | Zoca80 (OQ377889) | 92.59 | *Melospiza melodia* | DAB*417 (MH671019) |
|  | Zoca81 (OQ377890) | 90.48 | *Melospiza melodia* | DAB*332 (MF197795) |
|  | Zoca82 (OQ377891) | 89.47 | *Melospiza melodia* | DAB*332 (MF197795) |
|  | Zoca83 (OQ377892) | 95.70 | *Emberiza calandra* | DAB*26 (MW741162) |
|  | Zoca84 (OQ377893) | 95.16 | *Emberiza calandra* | DAB*26 (MW741162) |
|  | Zoca85 (OQ377894) | 95.31 | *Melospiza melodia* | DAB*511 (MF197840) |
|  | Zoca86 (OQ377895) | 93.65 | *Melospiza melodia* | DAB*515 (MH671092) |
|  | Zoca87 (OQ377896) | 98.94 | *Zonotrichia capensis* | C16E1 (AB531665) |
|  | Zoca88 (OQ377897) | 94.74 | *Emberiza jankowskii* | Emja-DAB18 (KT751216) |
|  | Zoca89 (OQ377898) | 93.16 | *Cactospiza pallida* | D3BM2 (AB531504.1) |
|  | Zoca90 (OQ377899) | 97.88 | *Melospiza melodia* | DAB*518 (MH671095) |
|  | Zoca91 (OQ377900) | 93.65 | *Geothlypis trichas* | Getr-DAB*1010 (JX215079) |
|  | Zoca92 (OQ377901) | 100 | *Zonotrichia capensis* | C16E1 (AB531665) |
|  | Zoca93 (OQ377902) | 99.47 | *Zonotrichia capensis* | C16E1 (AB531665) |
|  | Zoca94 (OQ377903) | 94.18 | *Ramphocelus carbo* | C28E2 (AB531656) |
|  | Zoca95 (OQ377904) | 94.79 | *Melospiza melodia* | DAB*511 (MF197840) |
|  | Zoca96 (OQ377905) | 89.95 | *Melospiza melodia* | DAB*122 (KX264078) |
|  | Zoca97 (OQ377906) | 93.12 | *Melospiza melodia* | DAB*494 (MF197837) |
|  | Zoca98 (OQ377907) | 91.01 | *Melospiza melodia* | DAB*431 (MH671029) |
|  | Zoca99 (OQ377908) | 93.65 | *Geospiza scandens* | D2VM12 (AB531728.1) |
|  | Zoca100(OQ377909) | 94.18 | *Melospiza melodia* | DAB*41 (KX263997) |
|  | Zoca101(OQ377910) | 92.59 | *Geothlypis trichas* | Getr-DAB*952 (JX215022) |
|  | Zoca102(OQ377911) | 93.65 | *Melospiza melodia* | DAB*41 (KX263997) |
|  | Zoca103(OQ377912) | 91.67 | *Melospiza melodia* | DAB*62 (KX264018) |
|  | Zoca104(OQ377913) | 93.65 | *Melospiza melodia* | DAB*41 (KX263997) |

Table 3. Tajima’s Neutrality Test. Evolutionary analyses were conducted in MEGA11. *Abbreviations*: *m* = number of sequences, *n* = total number of sites, *S* = Number of segregating sites, *p*_s_ = *S*/*n*, *Θ* = *p*_s_/a_1_, *π* = nucleotide diversity, and *D* is the Tajima test statistic.

|  |  | ***m*** | ***S*** | ***p*_s_** | ***Θ*** | ***π*** | ***D*** |
| --- | --- | --- | --- | --- | --- | --- | --- |
| MHC-I | PBR | 48 | 32 | 0.444444 | 0.100146 | 0.134936 | 1.169218 |
|  | Non-PBR | 48 | 48 | 0.307692 | 0.069332 | 0.065614 | -0.185677 |
| MHC-II | PBR | 104 | 25 | 0.757576 | 0.145219 | 0.252269 | 2.187147 |
|  | Non-PBR | 104 | 100 | 0.641026 | 0.122877 | 0.150977 | 0.749874 |

Table 4. Number of non-synonimous substitutions (dN) compared to synonimous substitutions (dS) in the PBR and non-PBR of MHC-I and -II sequences.

|  | dN/dS all sequence | dN/dS non-PBR | dN/dS PBR |
| --- | --- | --- | --- |
| MHC-I | 0,88 | 0,36 | 3,8 |
| MHC-II | 2 | 1.58 | 2.53 |

Table 5. Best model of the associations among rufous-collared sparrow MHC aminoacidic allele diversity and latitude, year and location.

| MHC-I | |  |  |  |  |  |  |
| --- | --- | --- | --- | --- | --- | --- | --- |
|  | Parametric coefficients | | | | | | |
|  | |  |  | Estimate | Stdr. Error | z value | Pr(>\|z\|) |
|  | |  | Intercept | - 0.45 | 0.083 | 5.43 | < 0.0001 *** |
|  |  | | | | | | |
|  | Deviance explained = 0 %. Link function = Poisson | | | | | | |
|  |  | | | | | | |
| MHC-II | |  |  |  |  |  |  |
|  | Parametric coefficients | | | | | | |
|  | |  |  | Estimate | Std. Error | t value | Pr(>\|t\|) |
|  | |  | Intercept | 3.28 | 0.109 | 30.07 | < 0.0001 *** |
|  |  | | | | | | |
|  | Approximate significance of smooth terms | | | | | | |
|  | |  |  | edf | Ref.df | F | p-value |
|  | |  | Latitude | 5.06 | 5.83 | 4.67 | 0.00044 *** |
|  |  | | | | | | |
|  | Deviance explained = 28.6**%**. Link function = Gaussian | | | | | | |
|  |  | | | | | | |
|  | Signif. codes: 0 ‘***’ 0.001 ‘**’ 0.01 ‘*’ 0.05 ‘.’ 0.1'' 1 | | | | | | |

Table 6. Best model of the associations among rufous-collared sparrow infection status and latitude, year and location.

| *Plasmodium* | |  |  |  |  |  |  |
| --- | --- | --- | --- | --- | --- | --- | --- |
|  | Parametric coefficients | | | | | | |
|  | |  |  | Estimate | Stdr. Error | z value | Pr(>\|z\|) |
|  | |  | Intercept | -4.76 | 3.07 | -1.54 | 0.12 |
|  |  | | | | | | |
|  | Approximate significance of smooth terms | | | | | | |
|  | |  |  | edf | Ref.df | Chi.sq | p-value |
|  | |  | Latitude | 2.54 | 3.05 | 4.63 | 0.24 |
|  |  | | | | | | |
|  | Deviance explained = 23.4 %. Link function = Binomial | | | | | | |
|  |  | | | | | | |
| *Haemoproteus* | |  |  |  |  |  |  |
|  | Parametric coefficients | | | | | | |
|  | |  |  | Estimate | Stdr. Error | z value | Pr(>\|z\|) |
|  | |  | Intercept | -1.41 | 0.29 | -4.82 | <0.0001*** |
|  |  | | | | | | |
|  | Approximate significance of smooth terms | | | | | | |
|  | |  |  | edf | Ref.df | Chi.sq | p-value |
|  | |  | Latitude | 3.82 | 4.504 | 10.33 | 0.051. |
|  |  | | | | | | |
|  | Deviance explained = 15.3 %. Link function = Binomial | | | | | | |
|  |  | | | | | | |
|  | Signif. codes: 0 ‘***’ 0.001 ‘**’ 0.01 ‘*’ 0.05 ‘.’ 0.1'' 1 | | | | | | |

Figure 1. Amino acid sequences of exon 3 MHC-I (A) and exon 2 MHC-II (B) of the Rufous-collared sparrow. + = Amino acid residues under positive selection based on Datamonkey analysis. * = PBR positions based on human MHC (MHC-I: (Saper et al., 1991); MHC-II: (Brown et al., 1993). • = Amino acid residues under positive selection based on (Minias et al., 2018).

A


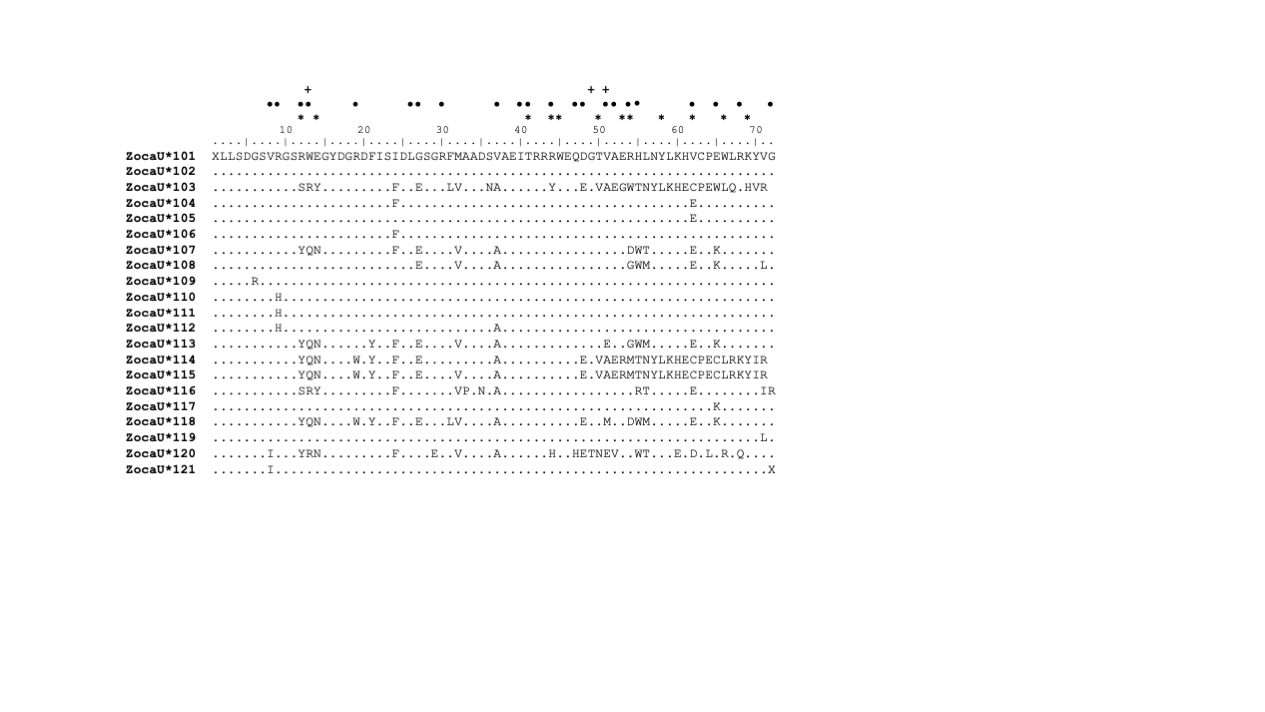


B


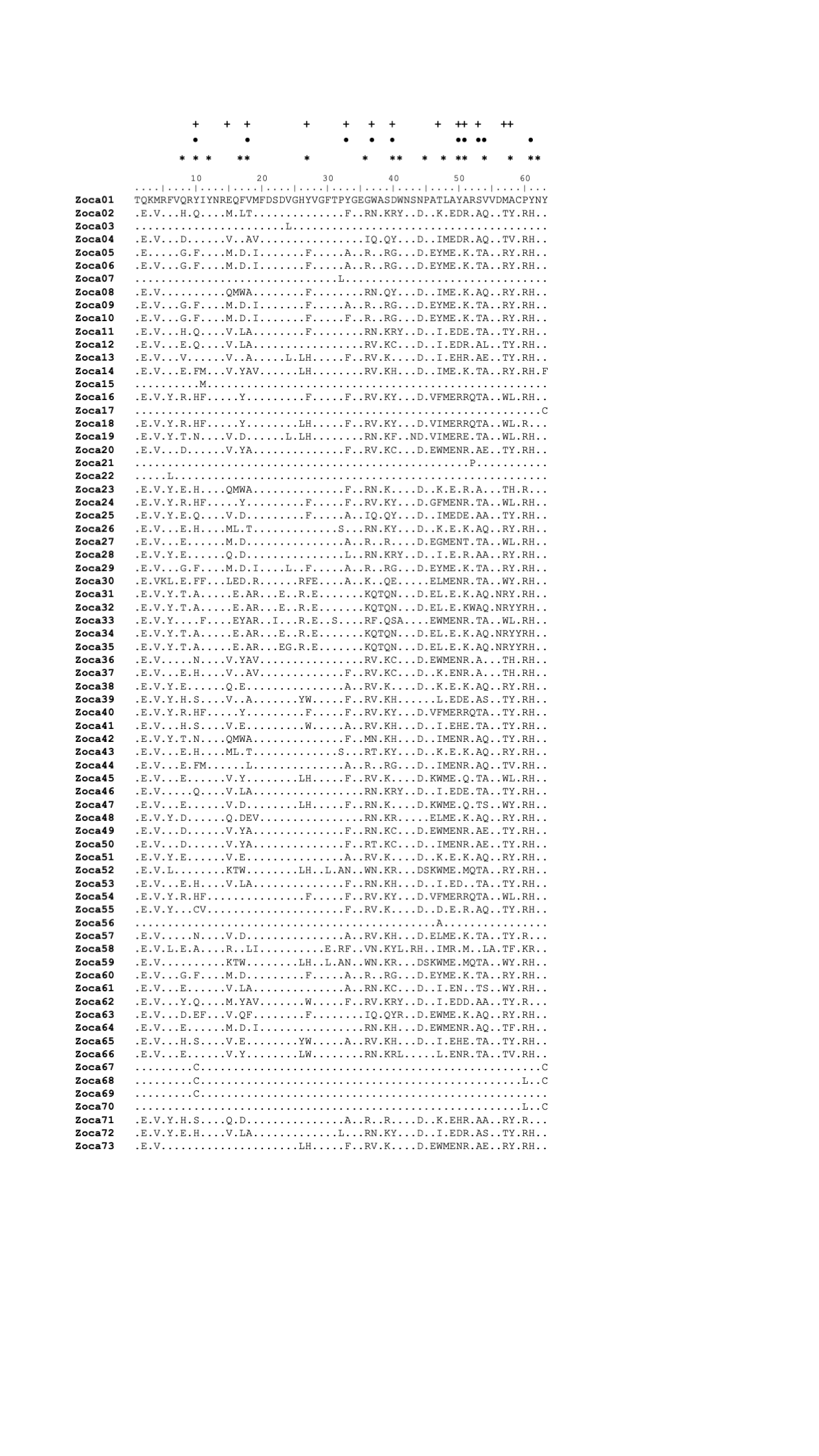

Figure 2. Maximum likelihood MHC-I (A) and -II (B) phylogeny calculated with PhyML. Best model of evolution was calculated with SMS. Branch widths represent aBayes node support. New alleles are highlighted in bold.

**A**

**B**

Figure 3. GAMM associations between *Plasmodium* (A) and *Haemoproteus* (B) infection status and latitude. Dot color intensity is directly correlated with number of individuals.

**References**

Brown, J. H., Jardetzky, T. S., Gorga, J. C., Stern, L. J., Urban, R. G., Strominger, J. L., & Wiley, D. C. (1993). Three-dimensional structure of the human class II histocompatibility antigen HLA-DR1. *Nature*, *364*(6432). https://doi.org/10.1038/364033a0

Minias, P., Pikus, E., Whittingham, L. A., & Dunn, P. O. (2018). A global analysis of selection at the avian MHC. *Evolution*, *72*(6), 1278–1293. https://doi.org/10.1111/evo.13490

Saper, M. A., Bjorkman, P. J., & Wiley, D. C. (1991). Refined structure of the human histocompatibility antigen HLA-A2 at 2.6 Å resolution. *Journal of Molecular Biology*, *219*(2). https://doi.org/10.1016/0022-2836(91)90567-P
